# Micro-drilling on shape memory alloys—A review

**DOI:** 10.1016/j.mex.2024.102968

**Published:** 2024-09-24

**Authors:** Kedarnath Chaudhary, V.K. Haribhakta

**Affiliations:** COEP Technological University, Pune 411005, India

**Keywords:** Micro-drilling, Chip formation, Burr formation, Micro-drilling tool, Shape Memory Alloys, NiTi – nickel titanium, Preferred Reporting Items for Systematic Reviews and Meta-Analyses

## Abstract

Technology like micro-hole drilling has progressed because of the machining industry's demand for high-precision micro components. Although micro drilling can be duplicated in theory, many challenges must be overcome before this becomes a reality. Micro-drilling with a thermal-mechanical interaction in the alloy is simulated in Abaqus. It looks at twist-bit modeling, material constitutive modeling, chip separation criteria, and element division. Focusing on work and tool electrode materials, hole specifications, micro hole types (through or blind holes), process parameters, performance measurements, and significant discoveries, this study presents a complete survey of the literature on spark methods for drilling microscopic holes. With an emphasis on spark erosion machining, drilling, and its modifications, this study seeks to aid researchers and academics by showcasing the potential of these methods to create extremely small holes. Microdrilling is critical for manufacturing miniaturized Nitinol components having applications in the medical field, robotics industry. Mechanical microdrilling offers a cost-effective and precise method over non-conventional ones.

Thus, the model may identify the drilling performance's key process parameters. They can also help operators choose micro-drilling-optimal machining parameters. Modeling micro-drilling software helps in shaping an alloy.

## Review Highlights

• Meta analyses.
• Micro drilling.
• Shape Memory alloys.

## Graphical abstract

**Figure:**
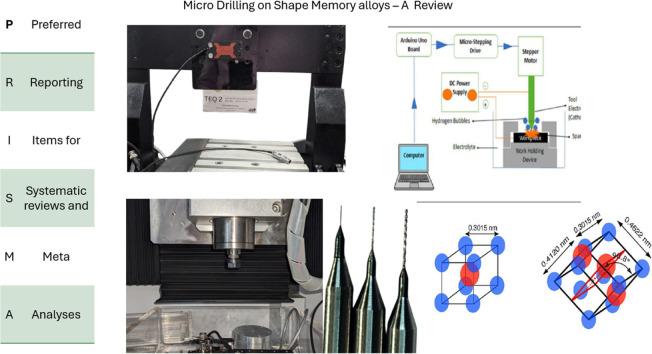

Specifications table

**Table:** 

Subject area:	Engineering
More specific subject area:	*Micro-Machining*
Name of the reviewed methodology:	*Prisma*
Keywords:	*Micro-drilling, Chip formation, Burr formation, Micro-drilling tool, Shape Memory Alloys, NiTi – Nickel Titanium*
Resource availability:	
Review question:	*What Micro-drilling?*
	*Which technique is used for the shaping of an Alloy?*
	*what components of Micro-drilling can be used to shape an Alloy?*
	*What can we use for designing the alloy with the designed shape and size by this technique?*
	*What is the performance of Micro-drilling for hard surfaces?*
	*How to measure drill bit life, hole quality, and damage mode during machining?*
	*What material is used for the micro-drilling process of shaping an Alloy?*
	*Strategies for Micro-drilling Mechanical Engineering used for completing the mechanism?*

## Background

The rise in Miniaturization with ever-changing customer needs and fierce market competition is prompting corporations to think creatively about how they produce goods. In addition, the widespread use of artificial intelligence, information, communication technologies, and other related technologies, as well as the demands for compactness, mobility, and adaptability in products, have forced the creation of new and novel paradigms in manufacturing. Recent years have seen a dramatic rise in the number of contexts in which micro-machining has proven useful. The majority of the goods in many industries that benefit from miniaturization are found in the realm of electromechanical systems, which are made possible via automation. Most micro-machined items are used in the automotive sector (for example, micro-jet holes of diameter 300 µm, depth >1 mm, and several holes >4 are commonplace in diesel engine injection nozzles), but biotechnology products, semiconductor devices, medical tools, aerospace, etc. have also benefitted from this [1]. Shape memory alloys have numerous applications in the aerospace and biomedical sector, due to their shape memory effect and superelasticity. It has been widely used in the smart wings of aircraft by replacing conventional heavy actuators [55]. Drilling micro-holes are of importance in the smart wings for attaching ribs, stringers, and other structural components to the wings and fuselage, ensuring superior strength and durability. SMA flexures are attached in the Boeing 777–300 ER commercial aircraft as a smart actuator by drilling holes [36]. SMA is used to fabricate micro-grippers in the robotics industry which also needs micro-holes on it [56]. For the implants made by SMA having alterable stiffness need micro-holes for the attachment purpose [57]. The advantages of its micro-machined characteristics have led to a proliferation of its uses. Injector micro-nozzles, for instance, have a significant impact on combustion, noise, emissions, and dependability in diesel engines. As a corollary, the downsizing of injectors for high-pressure diesel engines has improved the atomization, penetration, and spatial distribution of the injected fuel [2]. Valves and industrial torch tips are only two examples of the numerous products in the medical, aerospace, and other industries that depend on complex microfeatures*.*

## Method details

### Introduction

**Fig. 1:**
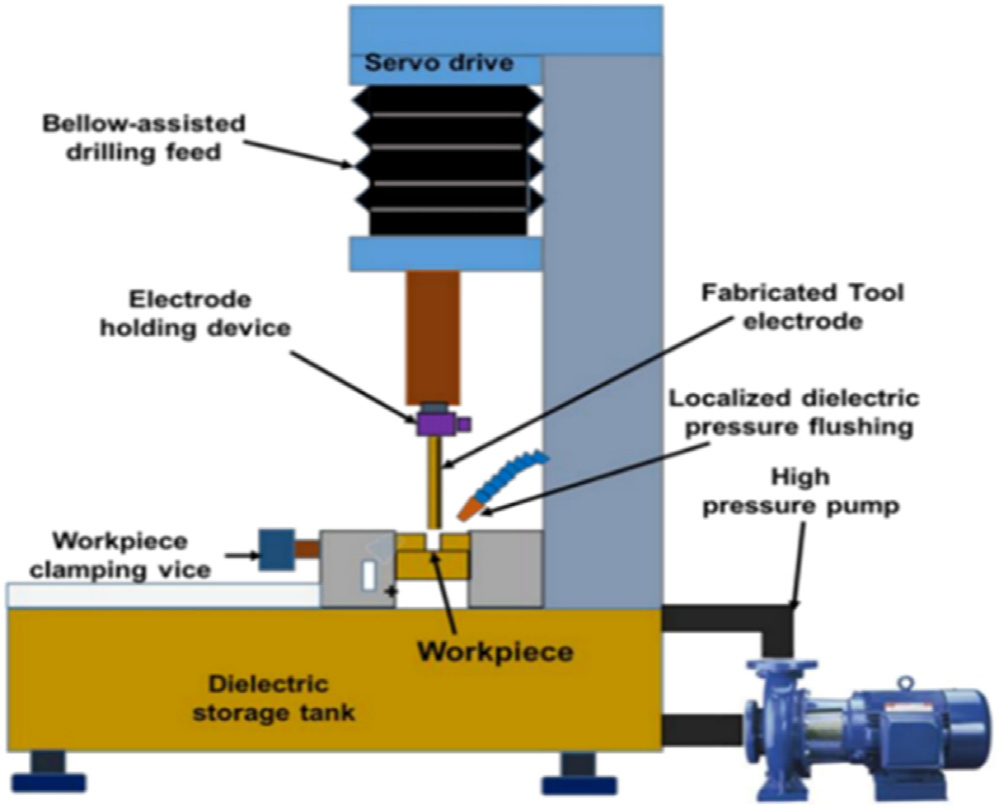
Micro-spark-erosion machining (µ-SEM) system for drilling [7].

Micro-drilling is a process that helps in creating holes in the range of diameter 0.05–2.5 mm, as defined by Siphinx—swiss microtool manufacturer [58]. The functionality of a product depends on details like the precision with which micro-features are fabricated, including their dimensions, surface polish, profile, and so on. A variety of micro-machining techniques exist, each with its own set of advantages and disadvantages. The machining forces in mechanical micro-machining are very high because of the size effect and thin tools, which results in frequent tools breakage, burr development, surface fractures, etc. [3]. The surface integrity and profile quality are both compromised during laser-based micro-machining. Due to its many advantages, electric discharge machining (EDM) has replaced other methods as the gold standard for micro and miniature-scale machining [4]. Since the EDM process does not include the mechanical action of a cutting tool or an abrasive tool, it can be used regardless of the hardness of the material making up the workpiece. The most recent developments and successes with the EDM technique have made possible what was formerly thought to be impossible in the realm of micro-machining. Several subsets have been developed for use in certain contexts, because of EDM's many advantages. These include micro-EDM, aeronautical EDM, semi-conductor EDM, medical EDM, and so on. The low discharge energy and high frequency of micro-EDM allow it to produce parts with the appropriate precision and surface polish when compared to traditional EDM. Selecting the right process parameters and operating within their range is crucial to micro-successful EDMs and efficient functioning [5]. In traditional EDM, the material removal rate (MRR) and the target surface quality are the primary considerations for determining the spark gap. To get the precision and low Electrode Wear Rate (EWR) required for micro-EDM, the spark gaps are maintained very narrowly. Micro-EDM results are sensitive to a wide range of variables, such as dielectric type, pulse profile, pulse current (e.g., shape, length, frequency, etc.), and so on. By reducing the spark energy, the EWR can be reduced. Producing both small and large features in micro-EDM (as is commonly necessary when making intricate 3D micro-profiles) is still a major cause for worry. This is because the electrode profile degrades due to EWR disparities between micro and macro characteristics. Drilling a blind hole with an electrode that wears at a consistent pace often produces a hole that is too tiny. In reality, because of a continual wear rate, the electrode diameter constantly decreases with increasing depth, resulting in an erroneous hole. It slows down the cleaning process, while a smaller spark gap improves precision and reduces Electromagnetic Wave Radiation in micro-EDM. As a consequence of this, the increased concentration of debris that is present among the electrode and the workpiece, along with the continuous feed, results in erroneous geometry in addition to poor surface integrity [6] Fig. 1.

To achieve high-quality results, micro-level machining is itself a complex process requiring great care and attention to detail. More precision is required if the micro-machining is performed on a challenging-to-machine material [6]. Shape memory alloys (SMA) based on the metals nickel and titanium (Ni-Ti) are one example of a challenging-to-machine material with many potential uses (biomedical, automobile actuators, micro-electromechanical systems, aerospace, etc.) [8]. Alloys with these characteristics stand out from the crowd thanks to their pseudo-elasticity, biocompatibility, corrosion resistance, and shape memory effect. Such features make them a great choice for a wide range of applications, but they also make them difficult to machine. Although Ni-Ti-based materials are challenging to manufacture, the shape memory effects add a layer of difficulty. In addition, the machining forces are high due to the size effect, and a thin tool is required for the conventional machining of micro-sized items. Consequently, micro-EDM is a viable alternative to conventional approaches for machining micro-shapes on SMAs. When doing micro-EDM, it might be difficult to determine what parameters to use and which solution is best since the electrode erodes during machining, which can hurt precision. Consequently, it is crucial to determine the optimal set of input machining variables for SMA machining. To better understand the influence of the incubation coefficient on the quality of microfeatures produced by laser-based micromachining of Ni-Ti-based SMA, single and multi-shot ablation threshold fluences were investigated. High-velocity milling of Ni-Ti-based SMA has its process parameters optimized [9]. It was discovered that a burr size of 15 m/min cutting speeds is suitable for achieving minimal machining forces. Micro-impact EDMs on hole quality while drilling tiny holes in nickel-titanium shape memory alloy [5]. Micro-EDM machining of Ni-Ti depends on SMA with Ti-6Al-4 V and the resulting surface properties. Ni-Ti-based SMA was shown to have a more refined surface quality than other types of SMA. In micro-machining, like micro-drilling and micro-milling, it might be difficult to achieve the appropriate dimensional precision, profile, surface quality, Material Removal Rate (MRR), Edge wear rate (EWR), etc. [10]. It all comes down to picking the right values for the process's variables. The process gets more difficult when more than one reaction has to be controlled simultaneously within strict parameters. It's important to note that each process parameter influences the outcome in a unique and often contrasting way. In other words, achieving peak performance from the micro-EDM process requires an optimization tool capable of determining the optimal settings for each variable. Acceptable micro-machined quality could be attained using tools like Multi-Performance-Characteristic (MPC) optimization. There are many different methods for optimizing MPC, and each has its advantages and disadvantages. While one MPC method could be perfect for a certain use case, it can provide misleading results when used for something else. Thus, it is crucial to choose the right MPC tools for a certain application to get the expected outcomes from micro-EDM. This problem has been addressed satisfactorily [11]. It demonstrated how using Taguchi's Grey Relational Analysis (GRA) in conjunction with Principal Component Analysis (PCA) can improve the precision of the machining. This approach synchronized Taguchi's experiment design, turned distinctive response data into similar normalized sequences by GRA without affecting its intrinsic essence, and linked numerous replies by applying a statistically calculated weighting of each response through PCA. Taguchi-based Grey-PCA is quantitative, whereas a typical MPC optimization approach is qualitative and relies on expert judgment. Inherent to the process of analyzing replies and performing statistical processing is the generation of weighting estimates. Furthermore, a thermoplastic injection-molded component's process parameters can also be determined using a fuzzy GRA approach. The wire-EDM processes for Al2O3 particle-reinforced composites were optimized for removal rates and surface roughness using GRA. In addition, both the Generalized Response Area (GRA) method and the Taguchi-based GRA could be used to optimize many turning process variables simultaneously. Both single- and multi-response optimizations have been the subject of a great deal of research. Micro-EDM micro-machining of Ni-Ti-based SMA is performed, however, there is a lack of data on the multi-response optimization of processing factors. Thus, the authors are doing this research to advance the state of the art. The purpose of the study was to find a way to enhance the precision of micro-EDM machining on a Ni-Ti-based alloy. Taper angle, overcut, and surface roughness is three quality parameters for which a technique has been created to optimize all of them. In the study, authors used a Taguchi-based grey relational analysis in conjunction with principal component analysis (Grey PCA) to examine the effects of three critical micro-EDM process parameters: voltage, capacitance, and electrode material [12].

### Conventional and non-conventional micro drilling process

#### Conventional micro-drilling process

In Mechanical micro-drilling process the tool makes direct contact with workpiece so the tool wear and machining forces are induced causing geometrical damage or tool breakage, makes it challenging [59]. Even though miniaturizing drill size poses various challenges that have a considerable influence on the micro-drilling process and output, macro-drilling and micro-drilling are essentially the same in many respects. Compared to micro drilling, macro drilling has a much bigger shank diameter, a thicker web, faster-rotating speeds, more vibrations, and other failure mechanisms [13]. Micro drills break much more often than regular drills. This is because drill bits with larger diameters usually wear out before they break, while micro drills break long before they wear out. The diameter of the shank is usually bigger than the diameter of the drill in the flute area because the spindle can only hold a drill with a certain minimum diameter. This is because the bit cannot be held securely in the spindle by the strength imparted by the micro drill's flute. In contrast to macro drills, where the shank diameter and cutting component diameter often remain constant, this is not the case here. In comparison to macro drills, micro drills have thicker webs, which increases the strength of the flutes. Moreover, to achieve a high aspect ratio, micro drills often have a bigger length-to-diameter ratio than macro drills. Typically, micro drills operate at a greater rotating speed, which further increases their fragility. Eliminating micro-drill failure is essential for maximizing output, enhancing quality, and maintaining worker safety. A greater amount of thrust and/or torque is produced, which is the primary cause of drill failure in micro drilling when more cutting power is applied [14]. The DRILL BREAKAGE PREVENTION (Drill strength>stresses existent in drilling) is thus achieved. SURVEILLANCE NETWORK (In-process preventing) IMPROVEMENTS TO TOOLS (Increasing mechanical strength) FACTORY CUTTING DOWN ON STRESS (of maximal values) REDUCING INCONSISTENCIES IN THE PROCESS (Unexpected rapid growth of loadings) Security of N, f Noise and vibration cancellations Minimizing chip build-up and extending tool life are two key concerns during chip removal. Reduce feed rate, pause drilling cycle, alter drill's design, use proper lubrication, and improve drill's performance and durability. Preventative measures against the breakage of micro drills Conducting a comprehensive analysis of the tool's mechanical properties, such as torque-torsion analysis, mathematical modeling, rotating and bending characteristics, tool wear mechanics, cutting force effects, and fracture features, can improve micro drilling performance and reduce the likelihood of breakage. The quality of micro drilling might range from one manufacturer to the next simply because each has its unique approach to production. The microstructure of the micro drill materials is another crucial factor in determining the quality and performance of conventional micro drills of every type. Even if two materials are made from the same basic ingredients, the grain distribution of their atoms can have a significant impact on the material characteristics, and hence, on the performance and durability of micro drills. The hardness of micro drills created from nano-sized particles is said to be significantly greater when compared to micro-range powders. It has been demonstrated that sintered materials with ultrafine grain sizes have exceptional flexural strength. Selecting appropriate materials with ultra-fine grain size and an optimal production procedure would be of considerable importance in the future, no matter the type of micro drills evaluated, as these factors directly impact the cost, performance, and durability of the micro drills. Advances in simulation technology have made it possible to model different micro-drilling settings to test out their impact and performance before it is fabricated. Powder modeling and molecular dynamics could be used to test whether or not ultra-fine micro grains improve micro drills' mechanical qualities. With the use of cutting-edge simulation technology, the feasibility of implementing a production process other than grinding and machining may be investigated. For instance, if the simulation of a forming procedure to produce micro drills using a direct powder solidification-extrusion forming technology proves effective, it might usher in a whole new era of micro-drill production.

#### Non-conventional technique

The unconventional methods discussed in this study have broad potential for use in today's fields of medicine, engineering, and the sciences. These technologies also cut, machine, and weld metal. Miniaturized perforation can only be performed by a select few of these technologies. As a whole, technical progress can either outpace or lag behind the need for micro drilling to satisfy present and future demands. Therefore, to meet the rising demands of micro drilling, serious study into creating these unconventional approaches is required. While laser micro drilling has the most adopters, it is not without its flaws. Poor dimensional accuracy, the creation of debris or a recast layer that must be removed, the existence of thermal residual stresses that cause micro-fractures in the hole, and a rise in manufacturing costs are all potential outcomes. Modifying the process parameters could remedy some of these drawbacks. Laser type, laser focal length, laser current, laser power, irradiation period, pulse width, air pressure, and gas condition are all factors in the process. Laser micro-drilling efficiency can be greatly enhanced by adjusting these settings. In addition to conventional methods, EDM micro drilling is a common non-traditional method of this drilling process. Any geometrical form or material of any hardness that is difficult to cut may be successfully machined using this technique so long as the workpiece being machined is electrically conductive. The removal of material throughout the process is still a difficult task to properly regulate, and as a result, the procedure is plagued with the issue of tiny fractures and poor surface quality. To improve the performance of EDM micro drilling, it is necessary to accurately manage the parameters of both the process and the system. The many benefits of Electrochemical Micro Drilling (ECMD), including increased material rate, low initial investment, faster production rates than EDM, and less environmental impact, have attracted a lot of attention recently. More research and development are needed to improve this technology's stray-elimination capabilities and insulation for its tools. The precision of the dimensions is still not as good as the standard one. Authors need further research to figure out how to fine-tune the system and adjust the settings so that can better cope with ECMD's drawbacks. Micro drilling using electron beams and ultrasonic vibrations are two relatively recent innovations. The primary goal of EBD's development was to facilitate the mass production of micro-holing in hard-to-machine or complex-shaped materials. A Computer Numerical Control (CNC) machine operated by the technology can be programmed to do many tasks. However, micro drilling by ultrasonic vibration is advantageous for several specialized purposes beyond only making high-quality tiny holes. Micro drilling techniques that don't already provide this option could be modified to produce holes of much higher quality thanks to this enhancement [15].

### Use of micro drilling in electrochemical

Ni-Ti SMAs, also known as Nitinol, rose to prominence due to the widespread usage it found in areas as disparate as telecommunications, automobile manufacturing, medicine, aerospace, etc. including civil industry [16]. The Ni-Ti SMAs can be used in a variety of medical fields, from Microelectromechanical systems (MEMS) to Thermomechanical Systems (TMS) for sensors and actuators to stent delivery to neurology implants to self-expanding stents to orthopedics and cardiology to drug delivery systems and implantable devices to eyeglass frames with Magnetic Resonance Imaging (MRI) compatibility [17]. Ni-Ti's high ductility and strong adhesion characteristic, together with its corrosion resistance and shape memory effect, provide challenges for traditional machining. In addition, the Ni-Ti SMAs' low thermal conductivity causes heat to concentrate on the tooltip, where it exhibits increased toughness, provides a higher Trigger Warning (TW), and encourages difficulties in conventional machining by causing excessive strain hardening. That's why it's best to use unorthodox machining techniques when working with SMAs [18].

Micro-holes in Ni-Ti alloys can be drilled using Electrical Discharge Machining (EDM), and electrical current-assisted machining (ECAM). Using the EDM technique and a copper drill bit, 5 mm by 5 mm by 5 mm blind squares holes were drilled in a Ni-Ti alloy. The electrical conductivity of the workpiece, Power dissipated in the OFF (POFF), gap current, and gap current were all taken into account as potential variables. According to the author's research, the gap voltage has a negative relationship with the MRR, meaning that a larger MRR could be attained with a lower gap voltage. With a higher gap current, the TWR also improved. Micro-Electrical Discharge Machining of Ni-Ti SMA for Drilling Micro-Holes. Performance metrics included Tool Wear Rate (TWR), overcut, circularity, MRR, taper angle, and Surface roughness (SR), while electrical parameters included voltage and capacitance for the tool electrode. It was determined that the micro-hole quality was highest at a lower voltage, whereas the overcut was primarily influenced by the electrode material [19]. Moreover, while drilling Ni-Ti alloy with micro-electrical discharge machining, the electrode materials were the most influential factor, followed by the voltage, in terms of performance characteristics such as SR, taper angle, and overcut [12]. Drilling Ni-Ti SMA using micro-EDM also detailed the movement of tools electrodes to the machined surface (MS), where new materials like Ni-TiO3 and TiO2 might serve as protective layers for the metal. In addition, a stable oxide layer was formed on the MS, which might allow for the use of traditional TiO2 coating on biomedical implants [10]. Previous works largely deal with machining by Numerically Controlled Mill (NCM) process involving drilling by EDM process, such as cutting, grooving, etc. Electron beam machining (EDM) has a worse surface quality finish while still maintaining a good machining rate because of metallurgical damage to the MS caused by the disadvantages of employing a liquid dielectric medium for sparking. Additionally, many constraints like as cavitation, spark damage, and electrolyte boiling have been documented in ECM. When thermal electrical discharges in the machining gap help the ECM process, the MRR may be as much as five or forty times higher than with Electrical Discharge Machining (EDM). This method is known as Electrochemical Arc Machining (ECAM). In addition, ECM tasks like monitoring radial overcut and delamination might be exhausting [12]. More development on the ECAM allows for the usage of hybrid NCM processes like ECAM for milling Ni-Ti SMAs. ECAM or SAEM was used to drill the microscopic holes in the electrically conducting substance (Sparc-assisted electrochemical machining) [20]. By using the SAEM method, a micro-hole may be drilled and electrochemically finished using the same machining equipment and the same settings [21]. Ni-Ti alloy electrochemical micromachining is best accomplished with ethanol electrolyte solutions containing 20 vol. The 40 g/L electrolyte solution was made by combining various quantities of ethanol and ethylene glycol and then adding sodium chloride to raise the solution's surface tension. Since ethanol may dissolve TiCl4 into the workpiece, it can also reduce the formation of oxide coatings [22].

Since, to the author's knowledge, no other work has explored the machinability of Ni-Ti SMAs by ECAM, this study used Electrochemical Arc Machining (ECAM) to drill tiny blind holes in Ni55.7Ti SMA. From a literature study, authors know that drilling tiny blind holes requires adjusting several process parameters, like tool rotation, voltage, and electrolyte concentration, as well as performance factors including MRR, delamination, and SR. The present research makes use of the Grey relational analysis-based Analytic hierarchy process (GRA-based AHP) in addition to the Generalized Reduced Gradient (GRG) optimized by Response Surface Methodology (RSM), both of which are examples of multiple objective optimization approaches. This method was used to find the sweet spot for enabling Ni-Ti SMAs' many potential uses. Many studies have assigned equal priority to all of the factors in an optimization model, while others that have used standard machining techniques, such as turning with Ti6Al4V alloy, have assigned varying weights to the variables depending on their relative value [23]. GRA-based AHP and RSM offered reliable means to boost performance characteristics in this non-traditional machining research because the AHP method guaranteed better results than equal weights.

### Micro-drilling with electrochemical arc machining (ECAM)

ECDM has undergone additional research and development, which has led to the discovery of an improved hybrid non-conventional machining technique. Combining ECM with EDM results in the creation of ECDM. During the ECM process, the workpiece serves as an anode, and the tool electrode functions as a cathode. Additionally, an electrolyte is employed as a conductive medium to form electrolyte cells together with the tool's electrode and the workpiece. These three components make up an electrolyte cell [24]. In the Electrical Discharge Machining (EDM) method, the material is removed using electrical sparks (discharges) when a quickly repeating current is used among the workpiece and tool electrode, which are separated by dielectric fluid. This causes the material to be eroded. Both the cathode and the anode are submerged in an electrolyte during the ECDM process, which results in the formation of an electrolyte cell-like ECM. During this process, the tool electrode is linked to the cathode, and an inert substance is employed as the anode.

Differences between ECDM and ECAM may be seen in the cathode and anode connections. In ECDM, the anode serves as a non-machining electrode; removing the anode for further connection with the workpiece allows for the ECAM procedure or the machining of electrically conductive materials. Discharges occurred between the workpiece and the tool in ECAM, but among the electrolytes and the tool in ECDM [25]. The electrolyte is employed as the dielectric in ECAM, which is a modification of the original EDM. Although it is common knowledge that Electrical Discharge Machining (EDM) is used for cutting micro holes, the use of ECAM for machining micro holes in electrically conductive materials is less well documented. Both steady and pulse voltages may be used to activate ECAM. ECAM can perform machining operations on metals such as drilling holes, cutting wire, turning, and finishing off rough edges.

**Fig. 2:**
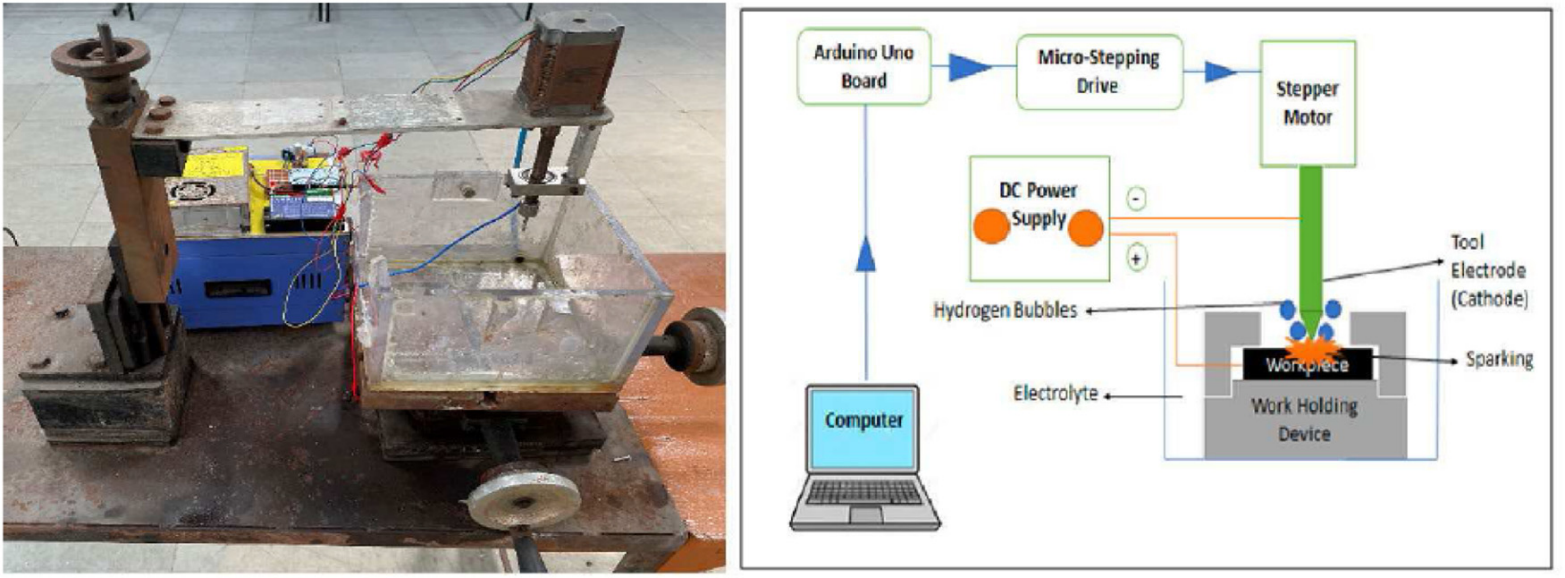
Micro-drilling with ECAM [26].

As illustrated in Fig. 2, during the ECAM process, the electrolyte is poured in among the anode (the workpiece) and the tool electrode, creating an electrolyte cell in which the anode is nearly completely submerged, and the tool electrode is isolated from the anode. Electrolysis takes place, resulting in the release of hydrogen gas at the cathode and oxygen gas at the anode when a voltage is applied across an electrolytic cell. An increase in voltage results in a greater production of gas. It is good to know that the form of the tool electrode is more condensed than that of the workpiece. As a result, gas will cover the tool electrode at a more rapid pace than it would cover the workpiece [26]. There is now resistance between the electrolyte border and the tools electrode as a result of the creation of the gas layer. An ohmic zone, representing ohmic behavior constrained to a voltage, formed when gas was ionized through the Townsend process after being forced to surround the tool electrode at a high enough voltage (U_lim_). The limiting current zone appears as a result of the formation of unified gas bubbles on the electrode surfaces and disappears after the critical voltage is reached when additional U_lim_ is supplied (U_crit_). A compact gas layer forms surrounding the electrode, indicating the formation of a transition area, when the applied voltage reaches U_crit_. The potential difference across the gas layer is said to have to be greater than a critical value for electrochemical discharge to occur. For this reason, if the applied voltage is made greater than U_crit_, an arc area will form in which a spark will fly.

### Machinability of shape memory alloys through different processes

Blair and Willie created a nickel-titanium alloy with 53 % to 57 % nickel content in the 1960s; this alloy produced deformed samples with strains ranging from 8 % to 15 %; after performing a thermal cycle, these alloys are prepared to return to their original structure. For this reason, shape-memory alloys have a brand name. One distinguishing feature of these alloys is that they may be heated and returned to their original state (to a heat elevated than their transformation temperature). One further thing that sets these alloys apart is their low yield strength, which makes them easy to shape and mold. Specifically, Shape-Memory Alloys (SMAs) are a class of compounds unto themselves due to their ability to maintain their original form after being bent. Plastic deformation of SMAs was seen at low temperatures, with the 'plastic' strain being acquired by increasing the temperature, a process known as the Shape Memory Effect (SME). To achieve a significant distortion when subjected to high temperatures, one must first disengage the affecting force.

Due to its exclusive mechanical and electrical properties, longer fatigue life, and strong corrosion resistance, Ni-Ti alloy, also identified as Nitinol alloy, is the most widely used SMAs and also the most expensive. The alloy of copper, aluminum, and nickel is one of the most widely traded metals in this sector. Additionally, SMAs can be created by alloying elements such as iron, zinc, and copper [27].

The advantages of shape-remembering alloys include their safety, resistance to pressure, and the fact that they can be used in zero-gravity circumstances while still maintaining clean, quiet, and spark-free working environments. As a result, it has found increasing usage in a wide range of disciplines, particularly in reciprocating applications (i.e., ones that constantly start and stop operating) like fire detection systems, clamping devices, and valves for refrigeration circuits. These reciprocating uses may be found in miniature forms, for instance, in electric motors that are used in very fine mechanisms [28]. Brackets, orthodontics, and medical guidewires are just some of the numerous medical applications for this versatile material. Also, SMAs were used in the aerospace sector and fixed-wing aircraft to power the wires (strings) that operate the ailerons, which do not have any hinges. Furthermore, a miniature F-18′s wings were twisted spanwise using a torque tube supplied by SMAs. SME is used to provide actuation in all of these configurations by resolving the shape change that occurs due to stresses.

**Fig. 3:**
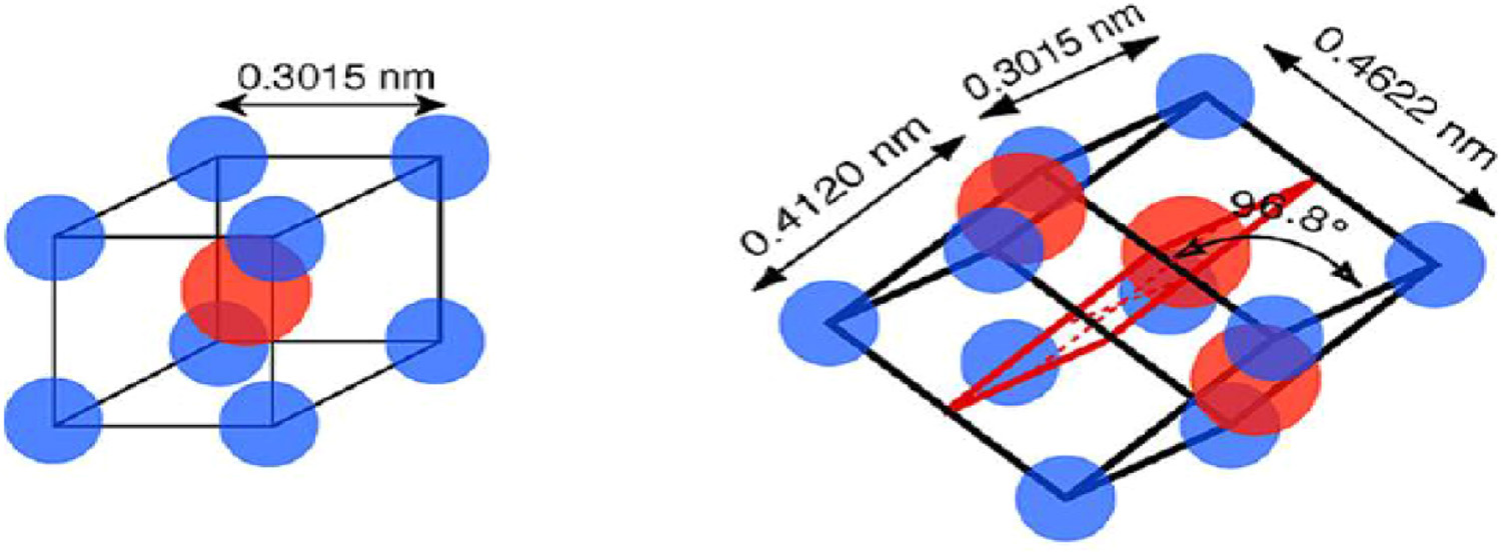
Nitinol austenite and martensite [29].

These properties of shape memory compound are the result of the martensite phase undergoing a reversible phase transition from a solid state to a solid state without undergoing any diffusion. It is a transition between a crystal-structured form known as austenite and another form known as martensite, which is less ordered. While cooled, the SMAs transition from the austenite phase, where they are when it is near relatively high temperatures, to the martensite phase. As can be seen in Figure, austenite has a cubic crystal structure, but martensite has a monoclinic crystalline formation. Both of these characteristics make austenite and martensite distinguishable from one another Fig. 3.

The transition from austenite to martensite is performed by a process known as distortion, which is defined as a change in the shape of the crystal structure caused by displacement. These changes take place inside the internal system of the material.

**Fig. 4:**
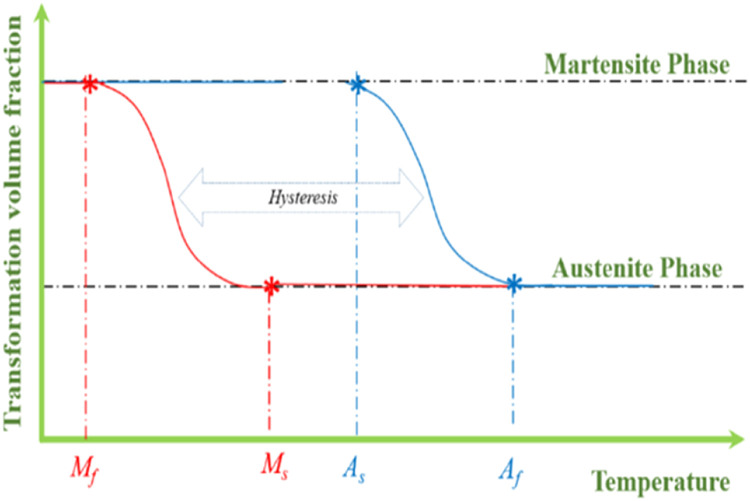
Phase transformation of SMAs [30].

In addition, austenite is more stable when subjected to high temperatures and low pressures, but martensite is more stable when subjected to greater pressures and lower temperatures. The definition of what it means when the temperature is high or low might be stated in greater detail in (Fig. 4). In addition, there is some time that elapses between acts. The temperatures at which austenite and martensite form are distinct from one another. The term “temperature hysteresis” is used to describe the transition from a martensite state to an austenite state that occurs at temperatures of 50 % [29].

SMAs can be characterized by one of four transition temperatures (M_f_, M_s_, A_s_, and A_f_). The transition from martensite to austenite occurs between the temperatures of A_s_ and A_f_, while the opposite transformation from austenite to martensite to austenite occurs between the temperatures of M_s_ and M_f_. In contrast, martensite is sometimes referred to as an ``inverse condition.'' This could lead to a change in the temperatures experienced at the transformation process's beginnings and endings when the primary and reverse transformation processes are repeated several times based on the property of the shape memory alloy. This phenomenon is referred to as functional stress, and it derives its name from the fact that it is associated with modifying the material's fine structure in addition to its operational qualities [30].

**Fig. 5:**
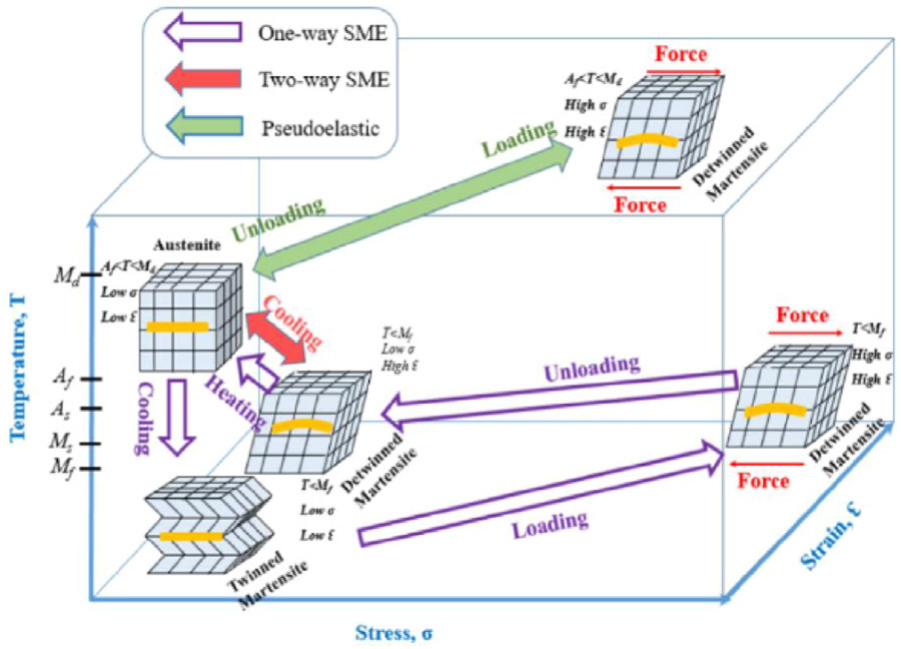
Explained are many strains, stress, and temperature SMAs [30].

As the temperature drops, the crystal structure of the austenite phase changes to become twinned martensite. This procedure would continue down to M_f_ once it has started from M_s_. The twinned martensite is distorted into a detwinned structure as a result of mechanical stress, although the fundamental form is preserved inside the SMAs. It is necessary to complete an additional two stages for it to restore its initial state. An austenite transformation requires first removing an external load and then heating the material to a high enough temperature to get it into an austenite state. A cycle is formed as a result of all these acts; hence, strain recovery varies from step to step. The building blocks of these measures are known as one-way SMAs. While the third method, which is referred to as two ways SME because it has a distinctive quality when heated to a higher temperature, one-way SMAs only exhibit a single initial form. However, with two-way SMAs, it was possible to establish two recoverable conditions: one of them in the austenite state, and the other with a martensite state (Fig. 3). Explain the different strain, stress, and temperatures SMAs (Fig. 5).

This section provides a concise summary of the many possible methods of operation that may be used to create SMAs, as well as their surface characteristics such as surface integrity, hardness, produced layer, and residual stress. How it reacts to different cutting fluids and equipment. However, conventional, non-traditional, and micro-operating all have an impact on the behavior and machinability of shape memory alloys, as do input characteristics including cutting velocity, feed rate, depth of cutting, coolant style, and coating type for submitted cutting tools.

### Applications of SMA

Many different uses may be found with SMA. It plays a key part in the Automotive sector. Common applications include electric generators that utilize waste heat to produce energy. SMA is utilized in a wide range of applications, from automated valves and miniature actuators to sensors and actuators, and even in noise-canceling components like solenoids. It is particularly useful in situations when a lighter weight will improve performance in a certain application. SMA is useful in the medical industry despite its lengthy reaction times, poor efficiency, tiny strain output, and substantial hysteresis. Intelligent Reinforced Concrete (IRC) is an SMA application that makes use of wires that are mixed in with the concrete itself. In such contexts, SMA may detect newly emerging fractures and aid in minimizing cracks of a larger magnitude. Biocompatibility is a major factor in why SMA has found such widespread application in endodontic files and orthodontic appliances. Bone fractures caused by SMA are also treatable [31].

#### Automotive applications

Increases in the use of actuators and sensors have made possible cars that provide greater comfort and safety than ever before, in addition to the must-have improvement in performance, therefore the scope of SMA has broadened [32]. Compared to electromagnetic actuators, SMA actuators are becoming more viable as automotive technology advances. Forcing heat in or out, many parts double as an active thermal actuators (for instance, clutches in the power train, temperature regulation in the engine, and carburetion), and as linear actuators [33]. Additionally, SMA also has uses in other fields, including aesthetics and aerodynamics, which is made possible by the SMAs' alluring changing abilities (active and adaptive structures).

#### Aerospace applications

SMA's special qualities make it particularly suited to the aerospace industry, where components must contend with tight spatial limits and heavy dynamic loads. Aerospace is a crucial application sector. These applications include but are not limited to, structural connections, actuators, release or deployment mechanisms, sealers, vibration-dampers, and the pathfinder application [34]. Another interesting way that SMA is utilized is in Variable Geometry Chevron (VGC), which is a device called a variable geometry chevron. Boeing was making it with SMA actuators, and the same thing has been put on a GE90-115B jet engine that is utilized in passenger planes. By making the chevron deflection as big as possible during takeoff, this device was able to cut down on noise. By making the chevron deflection as small as possible during the rest of the flight, it improved the efficiency of the cruise [35].

#### Robotic applications

Micro-actuators, or SMAs, are finding increasing use in a wide variety of industrial robots. There is a broad range of potential uses for biomechanically inspired SMA robotic technologies in the medical field. SMA has had significant usage in many other contexts, including the biological one. Increase the downsizing and functionality of the hardware components and improve the intelligence of the integrated systems (i.e., make them more autonomous, reliable, quicker, and smaller) [36]. Some of the many problems have been addressed, however, thanks to the careful choice of control methods, feedback sensors, and appropriate modeling approaches. The resistive feedback control, for instance, is great for little robots because it eliminates the need for extra sensors. .

#### Biomedical applications

Upon discovering SMA in nitinol in 1962, researchers began looking at the possibility of using SMA-based materials for dental implant applications. The first super elastic braces made from Ni-Ti alloy were described by researcher Andreasen in 1971, only a few years later. SMA-manufactured components have made important contributions to the biomedical field since their introduction in Minimally Invasive Surgery (MIS). This was made feasible thanks to the approval of the SMA's biological applications by the Food and Drug Administration (FDA) of the United States. These applications were created and put on the market in September 1989.

## Methodology

This Systematic Review (SR) follows the guidelines set out in the Preferred Reporting Items for Systematic Reviews and Meta-Analyses (PRISMA) statements for its methodology. Evidence-related systematic reviews and meta-analyses are compiled into the PRISMA statement. It is most often used for documenting analyses that evaluate interferences, but it may also be used to document systematic reviews that seek to do things like design, layer, or shape.

### Search strategy

A Systematic Literature Review (SLR) focuses on. Micro-drilling on shape memory alloys the primary report sources consist of four primary academic literature collections: Science Direct, IEEE, Springer, and Scopus databases. Study conduct SLRs to acquire information on related research in that field when researching a certain research issue or topic area.

Repeated database searches employing keywords to discover relevant scholarly content were undertaken up to the 26th of August, 2022. There was no time limit placed on the search for the key words in the abstract, which was searched alongside the other two titles and the subject (Scopus and others). A conference paper, article, or review was the only other acceptable document format, along with articles, reviews, proceedings papers, and bibliographies. The words that were searched for in both Scopus and Near are shown in Table 1. Several variations of the keyword's spelling were also checked. There were a total of eight files included in this group. See the keywords approach in Table 1.

**Table 1:** Scopus and other database search terms.

	Keywords
1.	What Micro-drilling?
2.	Which technique is used for the shaping of an Alloy?
3.	what components of Micro-drilling can be used to shape an Alloy?
4.	What can we use for designing the alloy with the designed shape and size by this technique?
5.	What is the performance of Micro-drilling for hard surfaces?
6.	How to measure drill bit life, hole quality, and damage mode during machining?
7.	What material is used for the micro-drilling process of shaping an Alloy?
8.	Strategies for Micro-drilling Mechanical Engineering used for completing the mechanism?

### Scrutinizing of paper for study

**Fig. 6:**
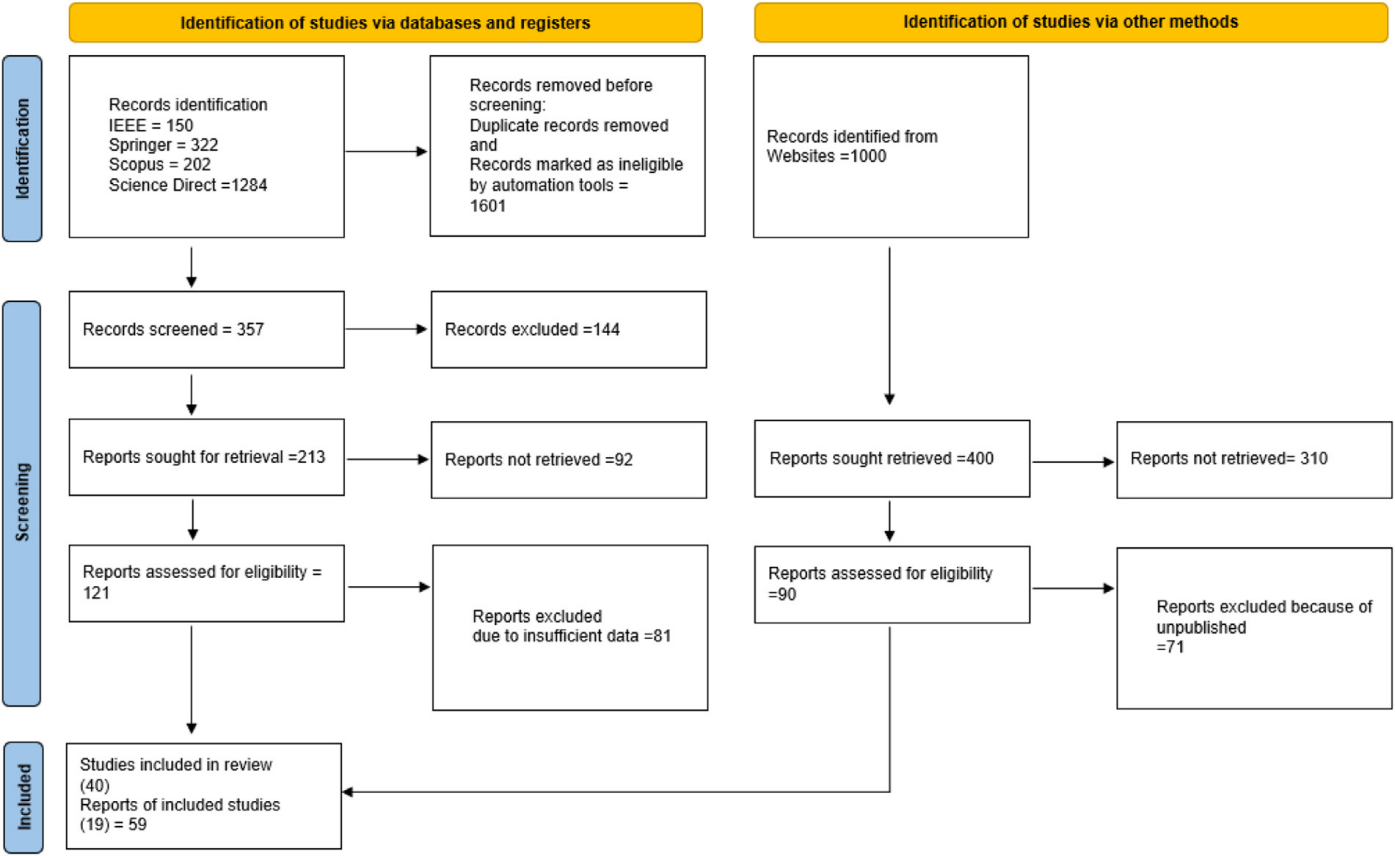
Flow chart for PRISMA-based SR of published article in data based.

There are four steps in the Primary Studies (PS) selection process: identification, admission, inclusion, and screening. Finding each relevant research is the first order of business; the first search returned 2958 hits. Conference proceedings were discovered after an exhaustive search of various databases and sources, such as full-text publications, science direct, springer, Scopus, and IEEE Xplore. The research was checked and examined for duplicates to get a final number of 54 studies. In the second stage, the author do a preliminary assessment by reviewing the article's title, keywords, and abstract. These two records, along with the ambiguous ones, were sent on for further assessment. An evaluation of the SR database is shown in Fig. 6.

All relevant articles and reviews must be found in the bibliography, which requires manual searching. The remaining paperwork was also scrutinized extensively. To decide which studies should be included and which should be left out of this systematic review, the author looked at their supplementary information and abstracts.

### Objectives of current study

Industries as diverse as electronics, aerospace, medical, and automotive manufacturing are all finding the growing need for micro drilling with diameters of a few microns to several hundred microns. Most of the credit for this goes to the widespread use of tiny products and equipment in these fields. There have been many novel methods of micro-drilling developed to satisfy this demand. However, no publication addresses all these micro-drilling techniques-and-on. The study delves into the state-of-the-art methods of micro drilling, organizes them into distinct categories according to their effectiveness in creating an alloy-friendly environment, zeroes in on the most new progresses and emerging movements, and lays out the requirements that would soon be necessary for this area of study. The study classifies both conventional and unconventional micro-drilling techniques used in modern applications. Drill bits in a twist, spade, D-shaped, single flute, compound drill, and coated micro drill shapes are commonly used in conventional micro drilling. Non-conventional micro drilling utilizes electrical, chemical, mechanical, and thermal approaches to achieve the same results when compared to traditional micro drilling. Here, it would compare and contrast several common micro-drilling methods with some less common ones to show how different kinds of shaping mechanisms can be used to best exhibit an alloy's potential and ensure appropriate stacking flexibility of micro-drilling's varied application options.

During the study's design phase, the three primary research questions were developed, and throughout the study, researchers are reviewed and evaluated. These are the inquiries we'll be making:

**RQ 1**: What are the methods to mold an Alloy into a different shape?
**RQ 2**: What are the novel fields to improve the precision that occurs in the micro-drilling process during shaping a mixture of two or more substances?
**RQ 3**: What are the limitations and research gaps of this existing technology?
**RQ 4**: Why do we use Different micro-drilling aspects and parameters regarding shaping in the research?

Firstly, To do this, a thorough analysis of the relevant literature was performed. To discover relevant articles published in the last decade, the author searched citation indexing databases and the internet of publications using tools like Sage, Emerald, Google Scholar, MDPI, Science Direct, IEEE Xplore, and Springer Link. In addition, an online inquiry was made to identify the best producers of wrist-mounted electronics. Results were examined using information obtained from white papers, manufacturer guides, and scholarly articles.

## Review of literature

This section describes the literature review which focuses on Micro-drilling on shape memory alloys.

Dutta et al. [37] Micro-electrical discharge machining, or -EDM for short, is one of the best ways to cut challenging materials precisely and accurately at the microscale. This work evaluates optimum micro-machining settings for low overcut and circularity error during -EDM of Nickel-Titanium (Ni-Ti) shape memory alloy using Response Surface Methodology (RSM) and Taguchi's Grey Relation Analysis (GRA)-based desirability function. Taguchi's GRA results show that the electrode material significantly affects the capacitance and discharge voltage responses. An ANOVA was performed, and the results showed that the electrode material had the biggest percentage influence (40.15 %) on both responses. The RSM-based desirability function methodology verifies the optimization outcome produced using Taguchi's GRA method. The final optimization result (electrode material tungsten, discharge voltage 80 V, capacitance 155 pF) matches the literature model (discharge voltage 80 V, electrode material tungsten, capacitance 355 pF) for improving micro-hole dimensional accuracy. The response surface plots also provided a comprehensive account of how overcut and circularity errors changed when process parameters were varied.

Das et al. [38] Authors are drawn to Ni-Ti Shape Memory Alloys (SMAs) due to their potential use in fields as diverse as aerospace, medicine, and robotics. This study looks specifically at using an Electrochemical Machining (ECM) procedure to machine (drill) Ni56Ti44 SMA at high temperatures. Different electrodes (brass, copper, and tungsten) and voltage (V), the current (I), and Inter-Electrode Gap (IEG) are included as controllable factors to examine how they affect machining quality aspects including the Rate of Material Removal (RMR), taper angle, overcut, and circularity error. Analyzes were carried out using a Box–Behnken design based on the Response Surface Methodology (RSM) technique to analyze machining properties parametrically. The utility approach also allows for multi-objective optimization of the desired answers at the output level. The outcomes of the desirability analysis utilizing RSM for RMR were shown to be superior, whereas those of the utility technique was superior for all other performance metrics. In this scenario, the Ni-Ti SMA at hand is best machined with a tungsten electrode, as shown by analysis of Scanning Electron Microscopy (SEM) micrographs.

Pliusys et al. [39] Most composite parts are made in a nearly finished state using just primary techniques. However, extra machining steps like drilling are often required. Delamination is perhaps the worst damage that could occur while drilling composites mechanically. The target of this work was to find a tactic to minimize delamination while micro-drilling thin carbon fiber-reinforced laminates using a 0.5 mm twist drill. The large size of the drill and the workpiece's varying composition make this a difficult task. Important machining input parameters were defined and ranked with the help of experiments based on Taguchi orthogonal arrays. An apparatus for holding the workpiece in place was designed and tested. There was a notable improvement in findings, with much less delamination damage being detected. Based on the data, spindle speed and drill point angle are the most influential factors in determining the severity of delamination at the point of entrance and exit. The component must be well-supported to withstand or be unaffected by the thrust force exerted by the drill as it nears the laminate's end. It is stated that the following conditions reduce delamination, reduce tool wear, and increase output.

Bhatta et al. [40] The need for tiny products in fields like physics, aerospace, medicine, and automobiles has increased the requirement for microscopic drilling with a diameter in the micron range. Drilling methods that use less material are being developed to keep up with demand. However, no paper has yet been published explaining, comparing, and contrasting all these common micro-drilling methods and highlighting the difficulties that they provide. This study studied the most up-to-date conventional methods and procedures for micro drilling, classifying them into distinct groups while also highlighting advancements, trends, and future needs in tiny drilling. Commonplace small-drilling methods for cutting-edge uses are categorized. Several different types of drill bits are used in standard tiny drilling, including twist drill bits, spade drill bits, D-shaped drill bits, single flute drill bits, compound drill bits, and coated small drill bits. A comparison of typical small drilling techniques is presented below. And required obstacles moon-faced to highlight the potential and adaptability of various tiny drilling methods.

Bharat et al. [41] metal matrix composites present a greater challenge during the machining process due to the presence of reinforced elements that are tough to cut when opposed to working with monolithic materials. Due to their superior high ultimate tensile strength, temperature resistance, strength-to-weight ratio, and great structural stability, metal matrix composites have been gaining prominence in the sphere of manufacturing industries across the world. The machinability of metal network composites has been enhanced by the use of non-conventional, conventional, and hybrid machining techniques. This work focuses on the analysis and research of various metal matrix composite machining cycles to achieve an improved outcome, and it is shown that hybrid machining plays a significant role in machining metal matrix composites as compared to other methods due to its superior machining capabilities. Non-conventional machining is essential to the achievement of the aim because hybrid machines are not widely available, and in addition, hybrid machines need to be operated by personnel who have the appropriate level of training.

Noor et al. [42] A sequential process was created that combines laser beam micromachining (LBMM) with EDM with the aim of micro-drilling to make use of the advantages of both techniques. A pilot hole is made using LBMM, and then the hole is refined using EDM. This method allows for a more consistent and effective machining regime, with increased processing speed (in comparison to pure EDM) and markedly improved hole quality (compared to LBMMed holes). According to the available data, there seem to be robust associations between the sequential process's input and output variables. However, there is no existing mathematical model that could map and predict all these output parameters from the input values at the same time. The experimental analysis revealed that the morphological condition of the LBMMed holes affects the different output parameters of the EDM finishing process. Due to the difficulty in predicting the results of a sequential process, a two-stage modeling approach based on Artificial Neural Networks (ANNs) was created. Outputs from the LBMM process were predicted using the first stage of the two-stage model, which considered a wide range of laser input characteristics. Further, in the second stage, LBMM predicted outputs (such as the first and last stages of the drilled hole, the recast layer, and the hazier parts of the hole) were used to make the final prediction of the sequential process outputs (i.e., EDM machining time, EDM stability measured in terms of the number of short circuits, and EDM machining tool wear). The entire sets of data for the model's output parameters (i.e., EDM time, short circuit count, and tool wear) were averaged to calculate the Root Mean Square Errors (RMSE). The average RMSEs for the parameters were determined to be 0.1272 (87.28 %), 0.1085 (89.15 %), and 0.097 (90.3 %).

Paul et al. [43] New materials and products often need the development of non-traditional micromachining technologies since traditional methods are unable to match their production needs. Micromachining techniques can be broken down into four distinct categories denoted by the source of the energy required to remove material: thermal, mechanical, chemical, and hybrid. To obtain the appropriate level of machining, hybrid methods combine two or more individual machining procedures. In the study, the authors would discuss some of the most important micromachining methods, as well as their respective material removal mechanisms and prominent application domains.

Oke et al. [44] Titanium-based components have a high price tag in part because of the high cost of machining. This is because there is a lot of waste from the workpiece and the tools wear out quickly. To bring down the price of titanium alloys, researchers have looked into doing away with the machining stage altogether, or at least towards finding the optimal settings for the machining process. The study suggested a summary of the conventional machining methods used for titanium-based components, because of the relative immaturity of the former. According to the nature of the titanium tool's initial contact with the workpiece, these procedures can be divided into two broad types. The two types of methods were labeled ``traditional'' and ``nontraditional'' machining. Numerous indications of machine-readiness were shown, most of which are directly related to the characteristics of these methods. This article discusses a variety of machinability indicators, including cutting forces, tool wear rate, surface roughness, chip formation, and material removal rate. However, tool wear, rates of metal loss, and surface roughness were highlighted. Improved machinability was also achieved by highlighting the crucial or optimal combination of factors. Many suggestions on where the research should go next are offered.

Mohammed et al. [45] The exceptional biocompatibility, shape memory effect, and super elastic characteristics of SMA based on Nickel-Titanium (Ni-Ti) make it useful in biomedical, automotive, and microsystem applications. Due to fatigue and previous pressures, these alloys are believed to be tough to cut, particularly with the technologies that have been around for a long time. Laser machining is one of the most efficient technologies available, when it comes to the processing of these alloys, particularly for applications involving microsystems. In this report, the authors describe the results of a comprehensive investigation into the impact of various process parameters on the microchannel machining in Ni-Ti SMA. A multi-objective optimization is performed to find the optimal input parameter values, to further ensure the desired output performance is achieved. The outcomes demonstrate how input factors have a major impact on microchannel quality. It was revealed that the microchannel's taper angle was significantly affected by the layer thickness. It was shown that layer thickness, scan speed, and scan technique greatly influenced scatter thickness and top-width error, although in different ways. The optimal solution developed by the multi-objective optimization that decreased taper angle and splatter thickness met the criteria of moderate velocity, maximum frequency, and lowest layer-thickness and track displacement.

Ranjan et al. [46] Mechanical, electrical, optical, ornamental, microfluidic, etc. devices all make use of micro-holes. For micro-drilling to achieve its goals of increased hole quality and productivity, however, tool wear and breakage must be tracked in real-time. The tool's health is tracked using data collected from a wide variety of sensors. In this investigation, the usefulness of vibration signals, cutting force signals, and their combinations as indicators of tool condition have been evaluated separately and in tandem. It has also been used to anticipate the quality of holes drilled using 0.4 mm micro-drills, providing insight into the most effective methods for keeping tabs on the state of the instrument being used. In addition, an Adaptive Neuro-Fuzzy Inference System (ANFIS) model was created for predicting hole quality based on characteristics extracted from these sensor inputs across many time domains and wavelet packets. Sensor characteristics in the wavelet domain of the vibration signal were combined to give the best prediction of hole quality. There was a strong correlation between the model's predictions and the actual outcomes.

Singh et al. [47] In recent years, there has been an uptick in the need for micro-holes to be drilled in materials that are otherwise difficult to machine, necessitating the employment of unconventional drilling techniques. Different unconventional drilling procedures are examined in this study, including electro-discharge, electrochemical, abrasive water jet, laser beam, and electrochemical discharge drilling. Every procedure has been scrutinized to determine its drilling mechanism, MRR/machining speed, and surface polish. These studies reveal that during unconventional drilling, the material is most often removed by evaporation, melting, chemical dissolution, and mechanical erosion. When compared to electrochemical discharge, electrochemical, and hybrid drilling methods, the understanding of laser beam, electro-discharge, and abrasive water jet drilling is far more advanced.

Liang et al. [48] It has been suggested that vibration hybrid-assisted micro-drilling and the ultrasonic cavitation approach may be used to enhance the micro-drilling performance of materials that are difficult to process, like stainless steel. To better understand the mechanism of ultrasonic cavitation and vibration hybrid micro-drilling, comparison tests between ultrasonic vibration micro-drilling (UVD), ultrasonic cavitation micro-drilling (UCD), hybrid micro-drilling (HD), and conventional micro-drilling (CD), are conducted. The effects of ultrasonic cavitation and vibration on stainless steel during the micro-drilling process are then analyzed taking chip morphology. According to experiments, both CD and UVD yield chips in the shape of a narrow strip, with the chips in UVD being much shorter than those in CD. Most UCD and HD chips, however, are quite short and fragmented, measuring just tens to hundreds of microns in length. While compared to CD, UVD, UCD, and HD all have lower thrust forces and micro-hole roundness errors when drilling to the same number of holes. HD has the lowest values of these three. High-Definition (HD) has the least amount of tool wear compared to the other three procedures. As a result, HD can substantially lower the thrust force and lengthen the life of the tool, while also improving the chip-breaking ability and resulting in the highest quality micro-hole processing possible. It is shown that the micro-drilling performance of stainless steel may be greatly enhanced by using an ultrasonic cavitation and vibration hybrid approach.

Dash et al. [49] Robotics, sensors, actuators, energy conversion, the medical field, aerospace technology, and more all make use of Shape Memory Alloy (SMA). It has been shown that traditional machining (where the tool and work piece are in contact) of SMA outcomes in poor surface polish, increased cutting force, and more tool wear owing to heat generation altering the material's characteristics and form. As a consequence of this, non-contact, non-conventional machining technologies, such as laser, Electro-Discharge (EDM), and Electrochemical (ECM) machining, are now required to machine SMAs. Although non-traditional methods provide several benefits over conventional machining, including higher machinability, each of these procedures also has several restrictions that must be considered. This study intends to provide a concise overview of the difficulties and potential benefits associated with the traditional and non-traditional machining of SMAs in general and Ni-Ti (Nitinol) in particular. The discussion has centered on the input parameters and performance measurements related to the machining of SMA material that has been described in the literature. In addition to that, an assortment of several optimization strategies used in this dominance for optimal forecasting reactions has been condensed.

Nishanth et al. [50] This work is a comprehensive guide to the numerous standard and non-traditional methods used to mill Ni-Ti-based alloys. The primary focus of this evaluation is on the uses of different alloys based on Ni and Ti. After introducing many common machining techniques, the authors go on to talk about the challenges of dealing with these alloys in the usual sense. Moreover, a variety of unconventional machining techniques, including cryogenic, ECM, EDM, UAT (Ultrasonic Assisted Turning), WJM (Water jet Machining), etc., have been discussed. The machining process's varied reactions are highlighted, including the rate of material removal, the roughness of the machined surface, and the rate of tool wear. Here, the authors give a synthesis of findings from several academics and studies on this subject. As result, the study has been wrapped up by highlighting several important points that were brought up throughout the review process.

Mehta et al. [51] Materials that are thought to be difficult to process include SMAs. The machinability of SMAs is covered in detail, and cutting-edge, environmentally friendly methods for machining SMAs are discussed in depth in this section. It starts with an overview of machinability, problems of SMAs, and potential solutions, and then moves on to explore the execution of machining methods and procedures to promote machinability. This section ends with a summary of the chapter's findings as well as a look forward at some potential future research capabilities including the machining of SMAs. The laser beam, Electric discharge, and abrasive water jet machining will get most of the attention throughout this chapter about the more sophisticated forms of machining that can be performed on SMAs. In contrast, the conventional turning, milling, drilling, and grinding of SMAs are discussed in the section on sustainable machining. This part emphasizes the application of modern cooling and lubricating systems. This study also covers certain features of current experimental research that was undertaken on the MQL-assisted turning of Ni-Ti shape memory alloy.

Venkatesan et al. [52] The production of holes on a smaller scale on a variety of different work materials might well be accomplished by a technique known as micro drilling, which is a sophisticated and accurate method. Superalloys play a significant part in the rapidly expanding area of engineering that is taking shape today because their principal qualities become more pronounced at higher temperatures. The purpose of this research was to compare the effectiveness of untreated and cryogenically treated drill bits during high-speed micro-drilling operations on the super alloy Inconel 800. The purpose of this was to learn how the various treatments affected response metrics such as material removal rate, thrust force, surface roughness, and temperature. Experiments are performed while taking into account the tool's diameter, feed rate, and spindle speed as process factors. These aspects of the process are adjusted by three levels by the Taguchi L27 orthogonal array design. The best settings for a process could be zeroed in on with the help of grey relational analysis.

Sivaprasad et al. [53] The creation of micro holes in materials that are difficult to cut is a common use of the laser drilling method. Although this method is quick, the holes produced by it are often of worse quality because of the unreliable nature of the laser beam. As a result, constructing a higher-order model and conducting an analysis of the process are both essential. There is also a strong need to find the ideal mix of factors that would result in the best holes being produced without sacrificing either the productivity or accuracy elements of the process. This analysis intends to identify the optimal laser parameter combination that, when applied to an Alloy-X material, would produce the best holes. There were four inputs taken into account and analyzed (pulse width, pulse frequency, power, and gas pressure), with six potential outcomes. Deng's Similarity-based strategy was used to evaluate the performance index of the response, and the relative weights of each output were determined using the entropy weight method. It was discovered that the factor with the greatest effect was the pulse frequency, whilst the element with the least impact was the gas pressure. The performance indicator is modeled with the help of the Response Surface Methodology. The heat effects during machining are explained by the metallurgical tests that were done in the micro-hole. Table 2.

**Table 2:** Selected studies and their contributions.

Authors name [ref no.]	Technique	Remark
Dutta et al. [37]	Grey Relation Analysis (GRA)	The response surface plots also provided a comprehensive account of how overcut and circularity errors changed when process parameters were varied.
Das et al. [38]	Response Surface Methodology (RSM) technique	The result of the desirability analysis utilizing RSM for RMR was shown to be superior, whereas those of the utility technique was superior for all other performance metrics.
Pliusys et al. [39]	Carbon fiber-reinforced laminates	It has been reported that the following circumstances boost production, decrease the amount of tool wear, and decrease the risk of delamination.
Bhatta et al. [40]	D-shaped drill bits	Under are typical small drilling methods and needed barriers moon-faced to demonstrate their potential and versatility.
Bharat et al. [41]	Hybrid machining techniques	In addition, hybrid machines need to be operated by personnel who have the appropriate level of training.
Noor et al. [42]	EDM	In this study, the authors found that the various output characteristics of the EDM finishing processes are impacted by the morphological state of the LBMMed holes.
Paul et al. [43]	Non-traditional micromachining technologies	The authors would discuss some of the most important micromachining methods, as well as their respective material removal mechanisms and prominent application domains.
Oke et al. [44]	Conventional machining methods	This study discusses a variety of machinability indicators, including cutting forces, tool wear rate, surface roughness, chip formation, and material removal rate.
Mohammed et al. [45]	Nickel-Titanium (Ni-Ti)	The outcomes demonstrate how input factors have a major impact on microchannel quality.
Ranjan et al. [46]	Adaptive Neuro-Fuzzy Inference System (ANFIS)	Sensor characteristics in the wavelet domain of the vibration signal were combined to give the best prediction of hole quality.
Singh et al. [47]	Unconventional drilling procedures	These studies reveal that during unconventional drilling, the material is most often removed by evaporation, melting, chemical dissolution, and mechanical erosion.
Liang et al. [48]	Ultrasonic cavitation micro-drilling (UCD)	As a result, HD can substantially lower the thrust force and lengthen the life of the tool, while also improving the chip-breaking ability and resulting in the highest quality micro-hole processing possible.
Dash et al. [49]	Shape Memory Alloy (SMA)	An assortment of several optimization strategies used in this dominance for optimal forecasting reactions has been condensed.
Nishanth et al. [50]	EDM	As result, the study has been wrapped up by highlighting several important points that were brought up throughout the review process.
Mehta et al. [51]	SMAs	In addition, the study delves into some of the specifics of the recent experimental studies that have been conducted on MQL-assisted turning of Ni-Ti shape memory alloy.
Venkatesan et al. [52]	A sophisticated and accurate method	The experiments are carried out by considering the process parameters of the tool diameter, feed, and speed of the spindle.
Sivaprasad et al. [53]	Higher-order model	As a result, constructing a higher-order model and conducting an analysis of the process are both essential.

## Discussion

### Requirement of procedure used in the shaping of an alloy using micro- drilling

**Fig. 7:**
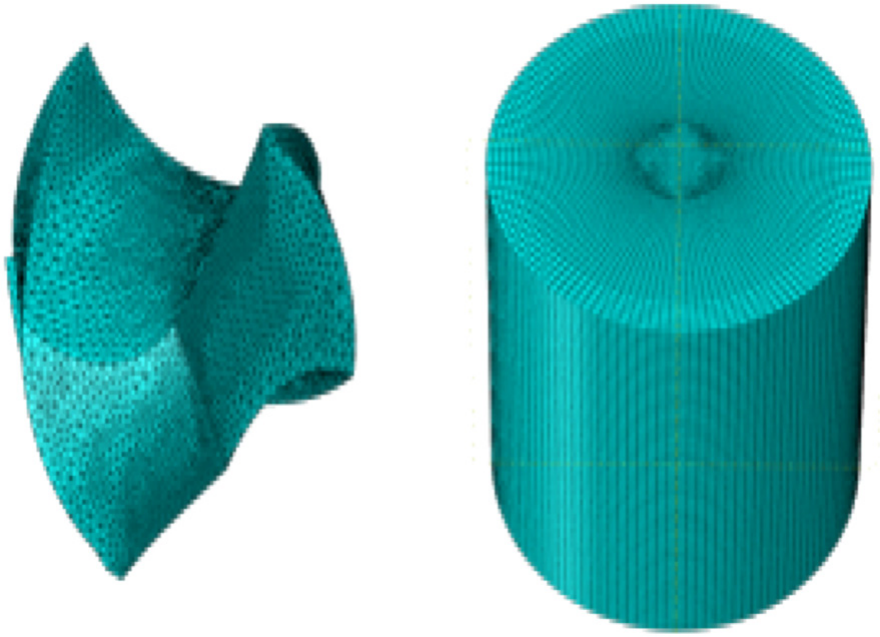
Cutting tool and work piece used [54].

Shaping an alloy using a drilling technique required various parameters to fulfilled the desired range. The few parameters are given below-Chip layout- The graphic demonstrates in Fig. 7 shows the stress cloud map that was generated because of the process that was followed for the simulation of micro-drilling. The chisel edge component makes the initial contact with the material of the workpiece during the process of micro-drilling for extrusion and friction, which ultimately results in elastic deformation. These steps are essential for the process to proceed. If the contact stress is higher than the material's yield limit, the material close to the surface would experience plastic deformation, and the lattice will become dislocated.

It can be difficult to keep the chip rotating in the same direction, because of the friction that exists between the micro-hole chip discharge groove and the hole wall. This is because of friction. One could consider this friction to be a drag on the system. Chips are difficult to create when the feed speed is less than 5 millimeters per second. Depending on how quickly the feed speed is, these chips may be broken up or debris-free. The chip discharge groove does not encourage chip discharge, because it is so easy to clog. The reason for this is that the plowing-cutting process plays a significant part in the whole cutting process, and it is this function that is responsible for the size impact that occurs. As a direct consequence of this, the outcome would be brought about. Both the size effect and the minimal chip thickness can be investigated through the ongoing expansion of the workpiece mesh as well as by comparison with the actual micro-drilling experiment. Both aspects can be investigated.

Force applied during layering and shaping- One of the most valuable elements to consider is the amount of cutting force that is delivered when it comes to determining how long a micro-drill would continue to function. As the chisel edge and the primary cutting edge gradually contact the workpiece during drilling, the amount of force applied to it grows steadily promptly after the drilling has begun. The main cutting edge starts to actively participate in the cutting process once the cutting time reaches 0.02 s. The maximum thrust force is reached, and the thrust force starts to oscillate at a steady level [54].

There is a lot of similarity between the changing laws of cutting force and torque. Torque increases gradually when the chisel's cutting edge and primary cutting edge gradually contact the workpiece and remains stable at a level that has been carefully reduced. Finite element simulations require continuous workpieces to be segmented before they can be processed into its constituent parts, wherein said separation serves to remove said constituent parts from said workpiece. Workpiece with elemental components. The sudden shock originates from the mesh element detaching from the workpiece. The thrust force will change, causing the thrust force and torque curves to oscillate visibly.

## Conclusion

In recent work, an effort was made to assess the influences of process parameters on surface performance measures while drilling alloy using the micro-drilling methods. This was done to better understand how these metrics can be affected. In addition, a response strategy was utilized to determine the mix of components that proved to be the most successful. Based on the results obtained from the experimental inquiry, one can draw the following conclusions:

1. Some sources claim that in addition to measuring the surface-induced attributes like cutting forces, surface roughness, and micro-hardness, the turning process can also be used to evaluate an alloy's machinability. Cutting speeds, feed rates, and depth of cut adjustments would allow for this.
2. The coating technique is used during the machining process to reduce the temperature, which in turn causes an expansion in the amount of time that the tools can be used. The lifespan of the tool can also be significantly increased by employing a technique that involves applying texture to the rake face of the instrument.

Therefore, the friction was reduced as a direct result of better lubrication, drilling torque, thrust force, and edge radius reductions were also made possible. As a result, the given parameters may be used to increase the drilling performances, the most crucial influencing factors. It can also be used in the real world to help operators choose optimal (or at least appropriate) values for the machining parameters that lead to an alloy with a better form thanks to the micro-drilling process. The software used to model the micro-drilling process can help with this.

3. This study has opened a large spectrum of investigation possibilities in conventional micro-drilling. This is due to the work that was done. The simulation may help to explain tool failures, surface integrity (including drilled hole surface roughness) issues, burr height, and chip characteristics.
4. The effect that cutting speed and drill diameter have on these machining properties can also be evaluated, and conventional micro-drilling techniques can be used to determine the optimal combination of process parameters to apply.
5. An investigation was conducted on the variation in a critical feed that takes place for various drill geometries because of the machining of various work materials. According to the findings of the study, the rate of feed was an important parameter that, when working with tougher materials like steel, should be decreased in comparison to when working with other types of soft materials to prevent the tools from breaking. The rate of feed is a key factor in determining how quickly the material is moved through the cutting tool.

The method that has been presented will be useful in selecting the operating parameters that are appropriate for the critical feeds that have been provided. The technology also has important uses in simulating the wandering motion of the drills during the initial phase and evaluating the positioning precision of the micro features. Both applications are noteworthy.

## Ethics statements

We all certify that this manuscript is not under consideration for publication in any other journal, nor has it been accepted for publication in any form, and no rights have been assigned to a third party.

## CRediT author statement

Kedarnath Chaudhary and Dr. Vikrant Haribhakta were involved in the conceptualization of the study. Kedarnath Chaudhary was responsible for data curation, while both authors contributed to formal analysis. Kedarnath Chaudhary wrote the original draft of the manuscript, and Dr. Vikrant Haribhakta reviewed and both authors edited it. Dr. Vikrant Haribhakta provided supervision throughout the study.

## Declaration of competing interest

The authors declare that they have no known competing financial interests or personal relationships that could have appeared to influence the work reported in this paper.

## Data Availability

No data was used for the research described in the article.
